# Caged Dexamethasone/Quercetin Nanoparticles, Formed of the Morphogenetic Active Inorganic Polyphosphate, are Strong Inducers of *MUC5AC*

**DOI:** 10.3390/md19020064

**Published:** 2021-01-27

**Authors:** Meik Neufurth, Xiaohong Wang, Shunfeng Wang, Heinz C. Schröder, Werner E. G. Müller

**Affiliations:** ERC Advanced Investigator Grant Research Group, Institute for Physiological Chemistry, University Medical Center, Johannes Gutenberg University, Duesbergweg 6, 55128 Mainz, Germany; mneufurt@uni-mainz.de (M.N.); shunwang@uni-mainz.de (S.W.); hschroed@uni-mainz.de (H.C.S.)

**Keywords:** mucin, polyphosphate, nanoparticles, dexamethasone, quercetin, ATP, SARS-CoV-2, human alveolar basal epithelial A549 cells

## Abstract

Inorganic polyphosphate (polyP) is a widely distributed polymer found from bacteria to animals, including marine species. This polymer exhibits morphogenetic as well as antiviral activity and releases metabolic energy after enzymatic hydrolysis also in human cells. In the pathogenesis of the coronavirus disease 2019 (COVID-19), the platelets are at the frontline of this syndrome. Platelets release a set of molecules, among them polyP. In addition, the production of airway mucus, the first line of body defense, is impaired in those patients. Therefore, in this study, amorphous nanoparticles of the magnesium salt of polyP (Mg-polyP-NP), matching the size of the coronavirus SARS-CoV-2, were prepared and loaded with the secondary plant metabolite quercetin or with dexamethasone to study their effects on the respiratory epithelium using human alveolar basal epithelial A549 cells as a model. The results revealed that both compounds embedded into the polyP nanoparticles significantly increased the steady-state-expression of the *MUC5AC* gene. This mucin species is the major mucus glycoprotein present in the secreted gel-forming mucus. The level of gene expression caused by quercetin or with dexamethasone, if caged into polyP NP, is significantly higher compared to the individual drugs alone. Both quercetin and dexamethasone did not impair the growth-supporting effect of polyP on A549 cells even at concentrations of quercetin which are cytotoxic for the cells. A possible mechanism of the effects of the two drugs together with polyP on mucin expression is proposed based on the scavenging of free oxygen species and the generation of ADP/ATP from the polyP, which is needed for the organization of the protective mucin-based mucus layer.

## 1. Introduction

On the cellular level, both macrophages and dendritic cells are the prime sensors of fungi, bacteria, and viruses that cause infection in humans. They are the starting points from where the innate as well as the adaptive immune defense and boosting machineries start [1]. The innate immunity, in the forefront of virus infiltration, activates the cells to build-up a chemical and also a physical barrier. The mucus at the interface to the environment overlays the surfaces of most epithelia. This layer, containing bioactive antimicrobial and antiviral supplements, forms a protective wall against microbes. Consequently, the mucus with its main structural components, the mucins, physically enwraps the viruses and concomitantly interacts with them by binding and inactivating the surface proteins of the viral envelope [2]. In addition, non-enveloped viruses such as picornavirus are encased by mucus [3]. Besides the adaptive immune system, the innate immunity forms one of the two defense arms that eliminate infectious microbes prior to the onset of disease. In 1796, a new area against infectious diseases started when Edward Jenner prepared an extract from a cowpox blister and scratched it under the skin of an eight-year-old boy, rendering him resistant against the pathogenic agent [4].

Acute respiratory infections (ARIs) are prevalent worldwide and together with diarrhea, are the leading cause of death, especially in developing countries [5]. Focusing on viruses, respiratory viral infections cause either endemic diseases, such as in tropical countries or in Asia-China, especially in children [6], but can also reach pandemic dimensions such as the coronavirus disease 2019 (COVID-19) [7]. The latter disease, caused by the severe acute respiratory syndrome coronavirus 2 (SARS-CoV-2), has been underestimated and was ignored at the beginning [8]. Both COVID-19 and influenza are contagious infectious diseases of the respiratory tract, which are spread via the airborne route in small (<5 µm) droplets where the virus particles can remain for long periods of time before they are inhaled into the respiratory tract [9]. Therefore, infection control based on these features is imperative for a successful prevention.

The respiratory viruses have to reach the epithelial cells via the bulky mucus layer, which is the first physical barrier that these viruses have to overcome. The epithelia of the different compartments of the upper respiratory tract are composed of specialized cells, the goblet cells, the basal cells as well as the ciliated cells and non-ciliated cells [10]. These cells are also framing the trachea and the bronchi. The histological diversifications are marked by specific mucins which are heavily glycosylated high-molecular-weight proteins [11]. The monomeric mucins, comprising in their central parts diversely compiled glycans, multimerize with their *N*- and *C*-terminal regions to a close-meshed, often gelatinous network.

The different mucins are divided into the group of secreted mucins and the tethered, cell surface-associated mucins [12]. The major secreted mucins are the five oligomeric, gel-forming mucins, such as *MUC5AC*, as well as two non-polymeric glycoproteins. The gel-forming mucins are layered outside of the epithelial cells, the periciliary layer, and form the epithelial lining fluid within the mucus layer. The directed motion of this layer is driven by the cilia protruding from the ciliated cells; the flow velocity of the mucus is 4–10 mm/min [11]. In contrast to *MUC5AC*, the tethered membrane-spanning mucins, such as MUC1, form the extracellular fluid into which the epithelial layer is embedded. The mucins are primarily secreted by the goblet cells.

Focusing on compounds that prevent SARS-CoV-2 infection, causing COVID-19, polyphosphate (polyP) has recently been found to bind to the receptor-binding domain (RBD) of the spike (S)-proteins that decorate the virus and associate with angiotensin-converting enzyme 2 (ACE2) receptor, an integral cell membrane spanning receptor of the target cells [13,14]. PolyP is a physiological inorganic polymer synthesized in any body cell, especially in blood platelets [15], and causes morphogenetic activity [16,17]. The polymer is highly accumulated in marine organisms, like in sponges [18] and in algae [19]. This polymer causes a complete inhibition of the binding of the RBD to the ACE2 receptor at a concentration of 0.1 µg/mL [14], a level that is much lower than the level of the polymer present in the circulating blood [15].

Similar to any other extracellular component, such as collagen, elastin or hyaluronic acid, or the extracellular organization of the β-amyloid [20], the extracellular mucin in concert with heparan sulfate is also structurally ordered [21]. An extracellular heat shock protein involved in this organization process is clusterin [22]. This molecule comprises a nucleotide binding motif [23] even though an ATP-independent functional activity has been proposed [24]. On the other side clusterin together with polyP abolishes the cytotoxic effect of β-amyloid, suggesting that polyP after its enzymatic cleavage and phosphotransfer by the alkaline phosphatase (ALP) and adenylate kinase (ADK), resulting in the generation of ADP/ATP, is providing metabolic energy which might be required for a correct folding of the β-amyloid [20]. In this context and on the basis of the finding that clusterin is a component of the extracellular airway cleaning squad [21] it can be proposed that clusterin together with the cell surface heparan sulfate receptor acts as an organizing component in the mucus layer, a process which is fed by ADP/ATP, generated from polyP during the ALP-mediated degradation [17].

For any virus that is entering the airway system, the composition of the mucins in the saliva and the mucus is crucial since it determines the clearance rate of the virus particles and the accessibility of the epithelial cells for the virus. The mucus/mucin layer, superimposing the periciliary layer that is covering the epithelial layer, has the major role to bind both to virus and to airborne particles and is composed of the secreted *MUC5AC*. In consequence, the soluble *MUC5AC* has its major role to drive the mucus clearance system to remove the virus/airborne particles out of the lung [25]. Surprisingly, it has been reported that in COVID-19 patients the levels of MUC1 and *MUC5AC* in the airway mucus are significantly elevated compared to healthy controls [26]. However, it has been proposed that *MUC5AC* facilitates general mucociliary clearance [27].

In a previous study, we used the human alveolar basal epithelial A549 cells [28], which display the ACE2 receptor [29], and showed that polyP strongly increases the gene expression for MUC1 and *MUC5AC* in these cells [2]. In comparison to A549 cells, the BEAS-2b cells do not show a considerable steady-state-expression of *MUC5AC* [30]. Searching for further natural products the marine secondary metabolite quercetin has been reported to regulate the expression of *MUC5AC* [31]. Quercetin (QCT) a secondary metabolite from the flavonoid group of polyphenols, is produced in a series of plants and also in the red marine alga *Alsidium corallinum* [32]. QCT has been described to suppress the expression of *MUC5AC* in HBE16 human airway epithelial cells. In these cells, *MUC5AC* is induced by the human neutrophil elastase, a serine protease which is secreted by neutrophils and present in high concentrations in patients with asthma and chronic obstructive pulmonary diseases [33]. In contrast, in the gastrointestinal tract QCT has been described to promote mucin secretion paralleled with an increase of the intestinal motility in rats [34]. This report has been confirmed by a concurrent work that revealed QCT to be an inducer of *MUC5AC* in intestinal goblet cells in vitro [35].

In the present study, QCT was encapsulated into nanoparticles (NP) matching the size of the SARS-CoV-2 virus particles, which measures 60 to 140 nm [36]. It should be highlighted that isoquercetin, the 3-*O*-glucoside of QCT, induces the ALP the enzyme that hydrolyzes polyP [37,38]. Dexamethasone, a synthetic anti-inflammatory acting corticosteroid, has been proposed as an ameliorating compound for COVID-19 patients, because it reduces the mortality in hospitalized patients with respiratory failure. Especially patients under supplemental oxygen or mechanical ventilation appear to benefit from this compound [39].

The presented data show that both dexamethasone and QCT, if caged into polyP NP, strongly induce the mucin gene expression. It is hoped that the NPs, enriched with dexamethasone and/or quercetin, contribute to a therapeutic intervention of the unbalanced mucin production in the airways system and amplify the natural antiviral immune defense in those epithelia.

## 2. Results

### 2.1. The PolyP-Based Dexamethasone and QCT NP

**Figure 1:**
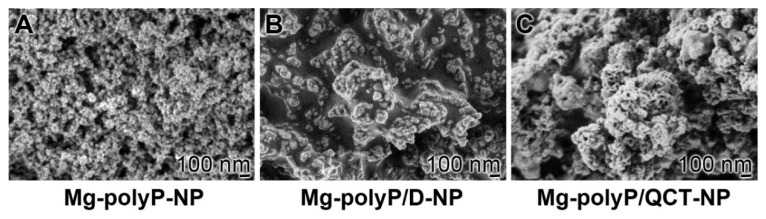
Fabrication of polyP-based quercetin and dexamethasone nanoparticles (NP); scanning electron microscopy (SEM). (**A**) The basic material, the NP, formed from MgCl_2_ and Na-polyP, are individually separated; “Mg-polyP-NP”. (**B**) Addition of dexamethasone 21-phosphate during the fabrication process from MgCl_2_ and Na-polyP gives raise to dispersed patchy, syncytium-like aggregates; “Mg-polyP/D-NP”. (**C**) Exposure of the “Mg-polyP-NP” particles to a QCT solution at the end of the preparation causes a fusion of the particles to larger patches, “Mg-polyP/QCT-NP”.

As initially described [40], the amorphous Mg-polyP NP were fabricated by precipitation at a superstoichiometric ratio (>2:1) between Mg^2+^ and Na-polyP, as reported earlier [41]. The particles formed had a diameter of ~74 ± 12 nm (Figure 1A). If during the fabrication, dexamethasone in the form of dexamethasone 21-phosphate disodium salt is added to the Na-polyP solution, the particles increase slightly in size to 100 ± 18 nm (Figure 1B). Under the conditions used, the particles also fuse together, forming patches of syncytium-like aggregates; “Mg-polyP/D-NP”. After addition of QCT to the “Mg-polyP-NP” fraction at a final stage of their preparation, the NP form larger fused patches (Figure 1C).

### 2.2. Size Determination of the Particles by Light Scattering

**Figure 2:**
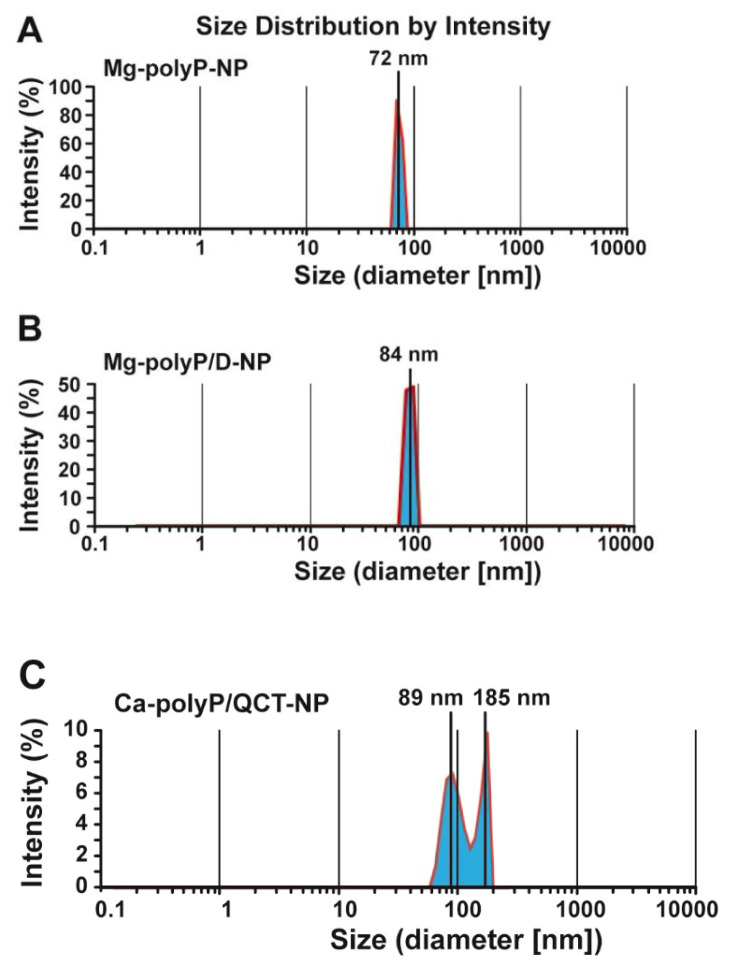
Size determination of the particles using dynamic light-scattering spectra of the Mg-polyP NP fractions (**A**) “Mg-polyP-NP”, (**B**) “Mg-polyP/D-NP” and (**C**) “Mg-polyP/QCT-NP”. At least 200 samples were measured for each preparation to obtain the size distribution. The experiments were performed with a Zetasizer Nano ZS90 at a scattering angle θ = 90°.

The NP of Mg-polyP have a globular size with a different degree of size distribution. The approach used, the dynamic light scattering measurements after dispersion in ultrapure water, allows for a more accurate determination of the size of the NP and their size distribution (Figure 2). The measurement of the “Mg-polyP-NP” sample amounted to 72 ± 18 nm; the sample shows only a small positive skewness with a δ of 0.1 (Figure 2A). The “Mg-polyP/D-NP” particles have a slightly larger average size of 84 ± 24 nm (Figure 2B). In contrast, the “Mg-polyP/QCT-NP” particles show a bimodal distribution with a smaller peak at 89 ± 32 nm and a larger one at 185 ± 39 nm (Figure 2C). From the latter data it could be deduced that the particles have the tendency to dimerize.

### 2.3. Interaction of Mucin with “Mg-polyP-NP”

**Figure 3:**
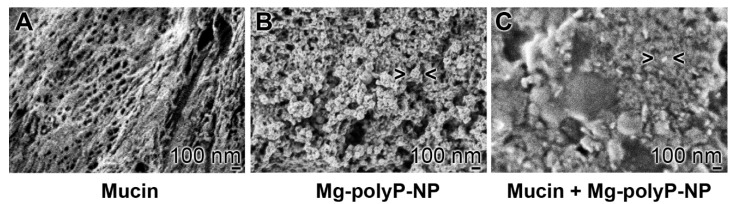
Solid surface formation of fibrillar mucin in the presence of “Mg-polyP-NP”; SEM. (**A**) Fibrillar mucin forming net-like structures. (**B**) A sample of “Mg-polyP-NP”. (**C**) Addition of “Mg-polyP-NP” to the mucin causes an almost continuous material with NP (> <) interspersed into the matrix.

In order to visualize the interaction of mucin with the “Mg-polyP-NP” a purified (enriched) mucin sample, originating for a commercial preparation was used (Figure 3A). Individual fiber-like structures are seen that form a net-like structure, due to their extensive oligomerization via disulfide bonds and additional weaker interactions [42]. In comparison, the “Mg-polyP-NP” particles with the average size of ~84 nm are shown (Figure 3B). If the particles are added to the mucin preparation, they fuse together and form a more homogeneous layer (Figure 3C).

### 2.4. FTIR Analysis of the Particles

**Figure 4:**
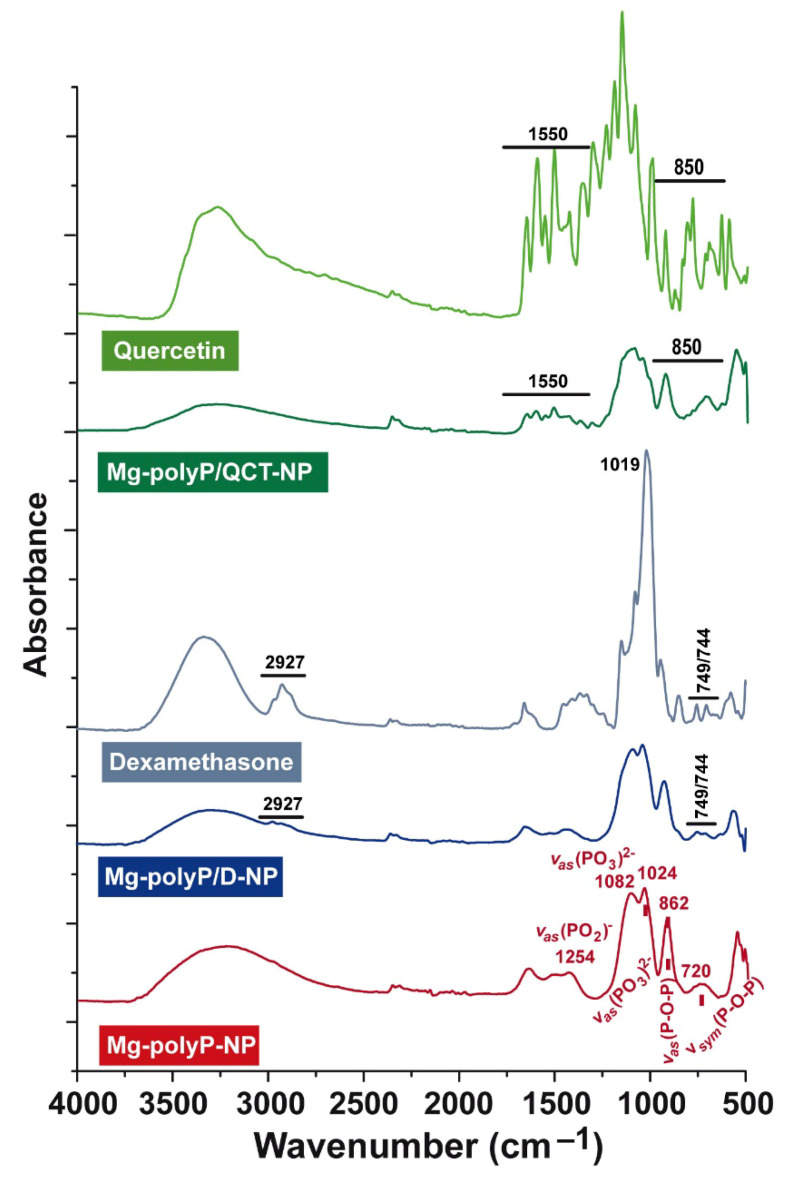
Characterization of the different materials by FTIR. Samples with a single component, either quercetin or dexamethasone, *versus* caged polyP-based NP. The spectra of the reference fractions “Mg-polyP-NP”, dexamethasone and quercetin were compared with the NP comprising the caged drugs dexamethasone, “Mg-polyP/D-NP”, and quercetin, “Mg-polyP/QCT-NP”. The characteristic signals of the reference samples are marked and also labeled in the caged fractions.

The FTIR analysis was performed with “Mg-polyP-NP” as reference material (Figure 4). Here, the typical signals for polyP are seen at: ≈720 cm^−1^ for the symmetric stretching vibration (ν_sym_) of P-O-P, 862 cm^−1^ for the asymmetric stretching vibration (ν_as_), ≈1024 cm^−1^ and 1082 cm^−1^ for ν_as_ [(PO_3_)^2−^], and 1254 cm^−1^ for ν_as_ [(PO_2_)^−^] [43]. The spectrum for “Mg-polyP/D-NP” has a comparable signal with the stretches at 2927 and 749/744 cm^−1^ reflecting dexamethasone [44]. The spectrum for quercetin is highly complex, especially around the vibrations of 1550 and 850 cm^−1^ [45]. These signals also show up in the “Mg-polyP/QCT-NP” sample.

### 2.5. Release Kinetics of Dexamethasone and QCT from the NP

**Figure 5:**
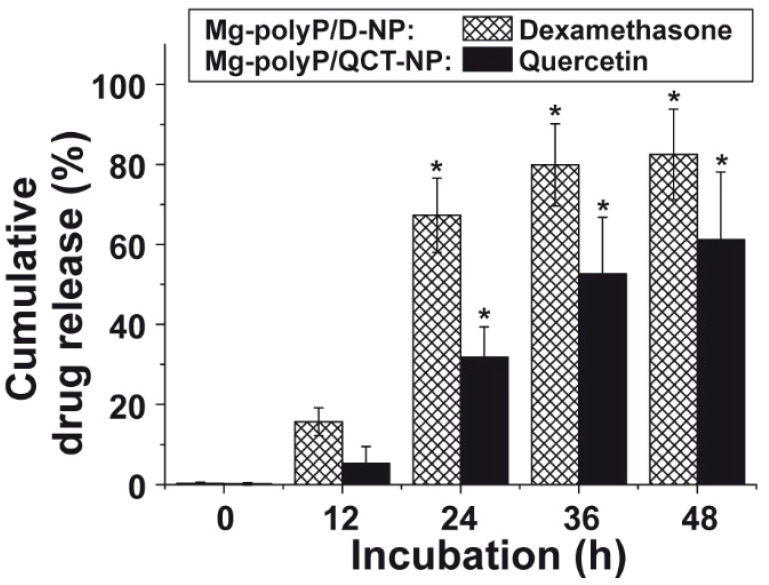
Release kinetics of dexamethasone and quercetin from the “Mg-polyP/D-NP” and “Mg-polyP/QCT-NP” factions in medium/serum. The released dexamethasone and quercetin were determined spectrophotometrically. The means ± SD of five independent experiments are given (*, *p* < 0.005 correlated with the release after 12 h).

As outlined under “Materials and Methods” the “Mg-polyP/D-NP” contained 2.63 ± 0.5 wt% of dexamethasone. Applying 20 mg of the particles and suspending them in 1 mL of medium/serum 15.7 ± 2.7% of the encapsulated dexamethasone was released into the surrounding milieu after 12 h. After 24 h and 48 h, the yield for the cumulated dexamethasone release increased to 67.3 ± 9.3% and 82.5 ± 11.3%, respectively (Figure 5).

The QCT content in the “Mg-polyP/QCT-NP” was determined with 0.8 ± 0.04 µg mg^−1^. Again, the release of the compound was determined in assay with 20 mg NP in 1 mL of medium/serum photometrically at 366 nm [46,47]. In comparison to dexamethasone, the kinetics was slower. While after 24 h only 31.0 ± 7.5% of QCT was released, this level increased to 61.2 ± 16.9% after 48 h (Figure 5).

### 2.6. Effect of Non-Capsulated and NP-Trapped Active Components: MTT

The influence of the free active components, Na-polyP, dexamethasone and QCT, as well as the NP formed on viability/growth of A549 cells was determined using the MTT assay.

**Figure 6:**
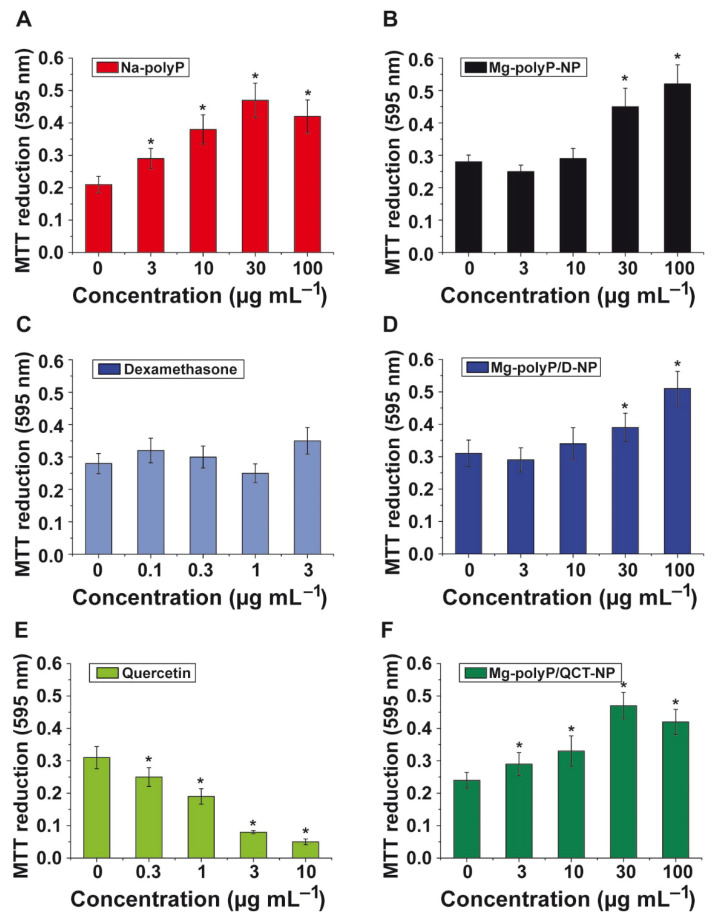
Viability/growth of A549 cells after incubation for 72 h with caged or non-caged dexamethasone and quercetin, as determined in the MTT assay. This reagent is assessing cell metabolic activity. (**A**,**B**) The cells were exposed to Na-polyP or “Mg-polyP-NP”. (**C**,**D**) A comparison of cell viability/growth between dexamethasone alone or the drug caged in NP; “Mg-polyP/D-NP”. (**E**,**F**) Effect of quercetin and “Mg-polyP/QCT-NP” on A549 cells. After incubation, the cells were subjected to the MTT color reaction, followed by a intensity determination of the formed formazan dye in a microplate reader at 595 nm. The data represent means ± SD (n = 10). The significances within an individual group (*, *p* < 0.01; against the values in the absence of the respective compounds) were calculated.

Na-polyP *versus* “Mg-polyP-NP”: The soluble Na-polyP added to the cell assays was co-applied with additional Mg^2+^ in order to compensate for the chelating activity of the inorganic polymer. This polymer, within the concentration range from 3 µg mL^−1^ to 100 µg mL^−1^ significantly increases the viability/growth of the A549 cells in vitro (Figure 6A). The NP-formulated “Mg-polyP-NP” likewise significantly stimulated cellular growth at concentrations higher than 30 µg mL^−1^ (Figure 6B).

Dexamethasone *versus* “Mg-polyP/D-NP”: As known from the literature, dexamethasone has only at high molar concentration >100 mM (39.2 mg mL^−1^) some toxic effects, similar to induction of apoptosis [48]. In the NP used for the studies here, 2.63 ± 0.5 wt% contributes to dexamethasone. Since after 24 h 67.3 ± 9.3% is released from the particles, a free drug level of 1.76 µg mL^−1^ (4.5 µM) is reached in the medium/serum at the highest NP concentration of 100 µg mL^−1^ (4.5 µM). The dose/response growth data show that at a particle concentration of 100 µg mL^−1^, even a significant increase of the viability by 60% is measured (Figure 6D). The free dexamethasone does not cause any significant change of the viability (Figure 6C).

Quercetin *versus* “Mg-polyP/QCT-NP”: The free QCT is cytotoxic in the in vitro assay with A549 cells (Figure 6E). Concentrations higher than 0.3 µg mL^−1^ significantly reduce the growth/viability of the cells. The compound was encapsulated together with polyP in NP reaching a concentration of 0.8 ± 0.04 µg mg^−1^. Addition of the NP at the highest concentration (100 µg mL^−1^, containing 0.08 µg of the quercetin compound) does not cause any decrease in the viability of the A549 cells, but instead results in an 80% stimulation of the viability (Figure 6F).

The data show that the components dexamethasone and QCT added to polyP and encapsulated into NP do not cause any inhibition of the metabolic activity of the cells by the MTT reagent. At the highest concentration of 100 µg mL^−1^ of drug-free NP, “Mg-polyP-NP”, a stimulation of the metabolic activity by 77% is measured, proposing that the activity supporting effect of polyP is not impaired by a co-presence with dexamethasone or QCT, under the conditions used here.

### 2.7. Effect of the PolyP-Based and Caged Drugs on MUC5AC Gene Expression

**Figure 7:**
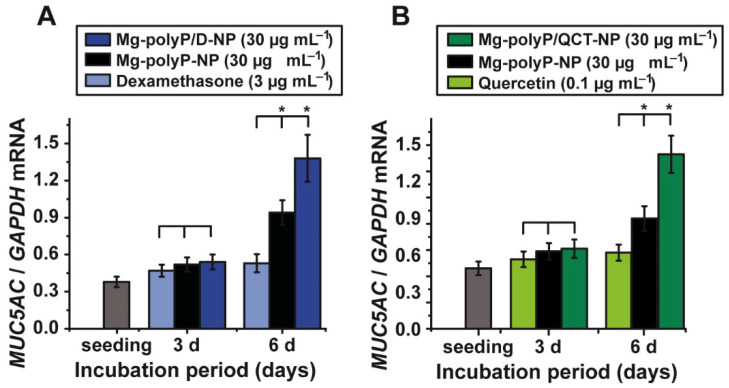
Gene expression level for *MUC5AC* in A549 cells after exposure to (**A**) 3 µg mL^−1^ of dexamethasone or 30 µg mL^−1^ of “Mg-polyP-NP” or “Mg-polyP/D-NP”, or (**B**) 0.1 µg mL^−1^ of quercetin or 30 µg mL^−1^ of “Mg-polyP-NP or “Mg-polyP/QCT-NP” during an incubation period of 3 d and 6 d, respectively. After the termination of the incubation, the RNA was extracted from the assays and subjected to qRT-PCR. Then, the expression levels were determined and correlated with the expression level of the house keeping gene *GAPDH*. Standard errors of the means (SEM) are indicated (n = 5 experiments per time point). Within one incubation time point (horizontal line) the significance has been calculated; *, *p* < 0.05.

The *MUC5AC* steady-state-expression study was performed to determine the effect of the drugs dexamethasone (Figure 7A) and quercetin (Figure 7B) in the free or the polyP-caged state. The compounds were exposed for 3 d or 6 d to A549 cells. Free dexamethasone was used at a concentration of 3 µg mL^−1^, while the NP formulations, “Mg-polyP-NP” and “Mg-polyP/D-NP”, were added at a concentration of 30 µg mL^−1^. As summarized in Figure 7A, 3 µg mL^−1^ of dexamethasone during the 3 d incubation period caused no significant difference in the steady-state-expression level for *MUC5AC*, compared to the drug-free “Mg-polyP-NP” or the caged “Mg-polyP/D-NP”, both added at the concentration of 30 µg mL^−1^. However, after the extended incubation period of 6 d the expression levels in assays with “Mg-polyP-NP” were 78% higher and for “Mg-polyP/D-NP” even 156% higher, compared to exposure experiment with 3 µg mL^−1^ of dexamethasone alone. This effect is remarkable, since the 30 µg mL^−1^ “Mg-polyP/D-NP” particles release during the 24 h only ~0.52 µg mL^−1^ of free dexamethasone (Figure 7A). This result indicates that the polyP-caged dexamethasone is a substantially stronger inducer of *MUC5AC*.

A similar amplification in the expression for *MUC5AC* is seen for the quercetin *versus* “Mg-polyP/QCT-NP” exposure studies. For the free quercetin drug, a concentration of 0.1 µg mL^−1^ was chosen, a level which is just below the lower toxic level of the drug with 0.3 µg mL^−1^ (Figure 6E). Again, the particle-caged formulation was added with 30 µg mL^−1^. During the first 3 d of incubation, the expression levels between quercetin alone and the NP formulations, “Mg-polyP-NP” and “Mg-polyP/QCT-NP”, caused no significant effect on the expression level (Figure 7B). After the 6 d incubation exposure, the level in the quercetin assay did not significantly change if correlated with the level at time 0. In contrast, the polyP-based NP, both “Mg-polyP-NP” and the caged “Mg-polyP/QCT-NP” significantly caused an upregulation of *MUC5AC* expression. The increase for “Mg-polyP-NP” is 36% and for the “Mg-polyP/QCT-NP” 133%. During this incubation period, about 0.02 µg is released from the 30 µg mL^−1^ of “Mg-polyP/QCT-NP”. In comparison, under the condition used, 0.1 µg mL^−1^ of quercetin alone showed no inducing effect on *MUC5AC* expression.

### 2.8. Immunofluorescence Analysis of MUC5AC

**Figure 8:**
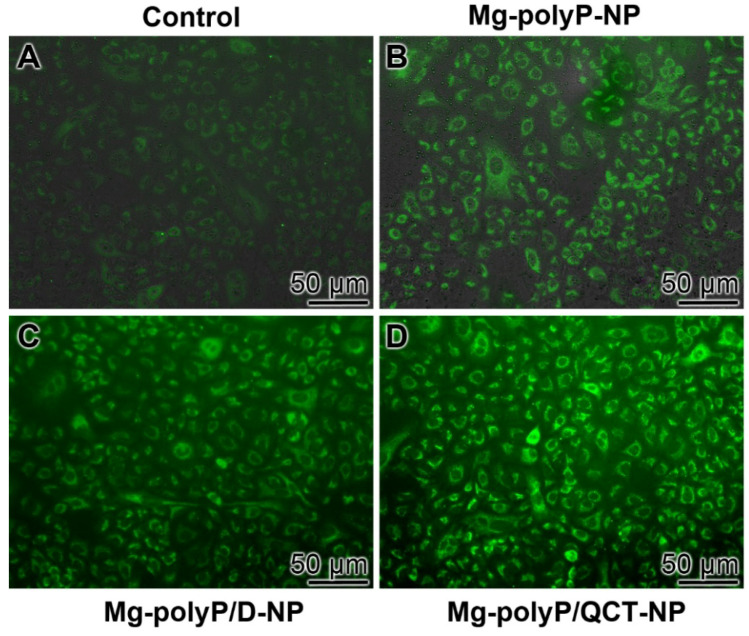
Expression of *MUC5AC* around the A549 cells after a 3 d incubation period (**A**) in the absence of a polyP component (control), (**B**) with 30 µg mL^−1^ of “Mg-polyP-NP”, or with either (**C**) 30 µg mL^−1^ of “Mg-polyP/D-NP” or (**D**) 30 µg mL^−1^ of “Mg-polyP/QCT-NP”. The cells were incubated with rabbit anti-human *MUC5AC* antibodies and then reacted with Alexa Fluor Plus 647 labelled goat anti-rabbit IgG secondary antibodies.

The change of the qRT-PCR expression data are further substantiated by reacting the expressed mucin with rabbit anti-human *MUC5AC* antibodies, followed by the visualization of the immunocomplexes with labelled goat anti-rabbit IgG secondary antibodies (Figure 8). In the controls, only a slight green fluorescence is seen (Figure 8A). Cells exposed to “Mg-polyP-NP” already show an increased reactivity (Figure 8B). However, the intensity of staining is substantially higher if cells are incubated for 3 d with “Mg-polyP/D-NP” (Figure 8C) or even with “Mg-polyP/QCT-NP” (Figure 8D).

## 3. Discussion

**Figure 9:**
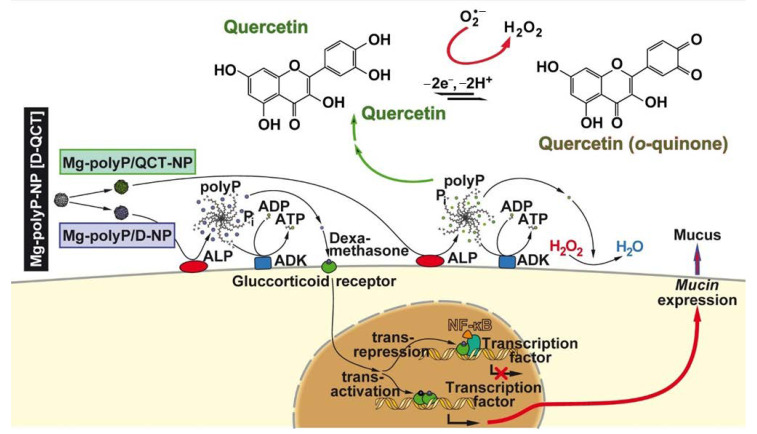
A sketched view of “Mg-polyP/QCT-NP” and “Mg-polyP/D-NP” metabolism on A549 cells *in vitro*. Both types of NP undergo hydrolysis and unfolding during the hydrolytic reaction though the ALP. During this process, dexamethasone is released from the “Mg-polyP/D-NP” particles and enters the cells where it binds to the glucocorticoid receptor. In the nucleus, the dexamethasone/receptor causes either *trans*-repression (*via* NF-κB), as *cis*- or even *trans*-activation. In turn, the gene(s) encoding mucin(s), MUC, are controlled, repressed, or expressed. In the same hydrolytic way, quercetin (QCT) is also released from the NP, which undergoes in the presence of free oxygen radicals a conversion to the *o*-quinone.

In the present study, it is shown that both the corticosteroid dexamethasone and the plant flavonol quercetin, a polyphenol, cause a positive action on the steady-state-expression of *MUC5AC*, especially if these compounds have been encapsulated into NP formed from Na-polyP and MgCl_2_. These two compounds are drugs. Dexamethasone is in medical use as an anti-inflammatory drug [49], used as a medicine that speeds the improvement of a sore throat [50], or as a flanking compound to counteract certain side effects during an antitumor treatment [51]. Basically the mode of action cannot be narrowed down to a single point of application, since its effect depends on the sensitivity and state of the glucocorticoid receptor [52]. Surely, dexamethasone interacts with the glucocorticoid receptors with a much higher affinity compared to endogenous cortisol [53]; the interaction of dexamethasone with the glucocorticoid receptor is prone to *trans*-activation, as well as *cis*- or even *trans*-repression [49]. In turn, dexamethasone can be involved both in a suppression and in a stimulation of gene expression (Figure 9). Dexamethasone has been shown to act as a catalytic radical scavenger during oxidative stress emerging during inflammatory or cardiovascular processes [54,55]. We did not approach the problem of a change of the glycosylation pattern of *MUC5AC* in response to dexamethasone or quercetin exposure. This alteration strongly affects the physiological interaction of the mucins within the mucus layer [56].

Quercetin has antioxidant activity by scavenging of reactive oxygen species followed by an elimination of oxygen radicals [57,58]. During this reaction, a reversible quercetin-quercetin semiquinone/quercetin *o*-quinone reaction occurs [59,60] during which the toxic effects of the quinone are eliminated [61], as shown in Figure 9. Quercetin is used as a drug to ameliorate adverse oxidant reactions by acting as anti-inflammatory, antimicrobial, antineoplastic, neuroprotective, and even anti-allergic drug [61].

The two drugs, dexamethasone and QCT, are caged into polyP NP. In this study, we used the “Mg-polyP-NP” particles. During the hydrolytic cleavage of the acid anhydride linkages in polyP, free energy is released, which is stored in biochemically usable ADP, which can further on convert to ATP via the ADK [17,62]. Both polyP and the starting compound phosphate are considered safe by the U.S. Food and Drug Administration. PolyP is used, due to its action to retain muscle moisture in fresh or frozen tissue, to improve meat texture, prevent microbial spoilage, improve flavor, and promote protein gelation [63]. In this context, it is important to mention that in the airways system, the mechanical strain, loaded onto the cilia of the airways system, increases the hydration, regulates the ATP production and releases and, by this, adjusts fluid secretion and optimizes mucus organization on top of the epithelial cells [64,65]. Interestingly ATP has been found to stimulate mucus secretion [66]. Since polyP produces ATP during enzymatic degradation by ALP and subsequent phosphorylation of ADP to ATP via ADK [17], it can be proposed that during this reaction cycle polyP controls also the synthesis of *MUC5AC*. In turn, this ATP generation from polyP, which is itself produced from ATP in the acidocalcisomes, will need a detailed analysis of the coupled reactions by metabolic flux analysis in the future, as outlined [67]. One way to analyze this pathway is by deuteronation, as exemplified for ATP synthase [68].

The present study shows that the cytotoxic activity of quercetin, above a concentration of 0.3 µg mL^−1^ (~1 µM), is abolished if this compound is caged into polyP NP. Applied together to the A549 cells, an over two-fold increase of the steady-state-expression of *MUC5AC* is observed. A similar effect is disclosed also for dexamethasone. While this synthetic corticosteroid does not cause a marked effect on cell growth, it strongly increases the expression of the *MUC5AC* gene if embedded into polyP NP. At present, the most plausible explanation for the strong effect of the two drugs on *MUC5AC* expression is a mutual influence of the components dexamethasone, quercetin and polyP on the reactive oxygen species load of the cells. This view is strengthened by the finding that during mucin, *MUC5AC*, synthesis, which is initiated by the epidermal growth factor, reactive oxygen species are formed that modulate the expression level of mucin genes [69]. Parallel with these reactions, the gene expression of the dual oxidase 2 enzyme (DUOX2) is upregulated. In turn, DUOX2, as a member of oxygen radical forming cell membrane NADPH oxidases, is a signal generating enzyme that induces the synthesis of mucin [35]. It can be assumed that this process is adjusted by the antioxidant activity of quercetin, which is increased after complex formation with certain divalent cations [70], including magnesium [71]. Mg^2+^ ions are released during enzymatic (ALP-mediated) degradation of the Mg-polyP-NP. The free radical scavenging activity of the quercetin-Mg^2+^ complex has been reported to be significantly higher than that of quercetin [71]. PolyP released during disintegration of the NP may also interact with other ions present in solution, forming a complex able to inactivate superoxide anion radicals via a non-enzymatic mechanism. For example, polyP may show antioxidant activity by complexing divalent manganese (Mn^2+^) ions that becomes oxidized to Mn^3+^, remaining bound to the polymer [72]. Taken together, the two medicinally applied compounds dexamethasone and quercetin, in concert with polyP as the caging polymer, adjust the suitable level of reactive oxygen species for an increased mucin gene expression. Surely, the potential exposure time of dexamethasone in the airways system would be short and perhaps not long enough to affect the proliferation of the cells. However, the effect of dexamethasone on the cell cycle phase requires further studies [73]. Again, the powerful technique of metabolic labelling, starting perhaps from [1,2-^13^C_2_]-d-glucose, might be a suitable way to follow [74].

The medical value of both compounds, dexamethasone and quercetin, is well established. In the present study, it is underlined that if the compounds are embedded into the polyP scaffold, a 50% release is measured after ~24 h. This delayed release is especially favorable for the growth-reducing function of quercetin, allowing an exposure of this compound for a longer period at biologically preferable concentrations.

A further notable aspect of the present data is that the polyP NP undergo together with mucin a coacervate-like transformation. This observation is remarkable since the coacervate form of polyP has been shown to be the biologically active form of the polymer [75]. Moreover, the coacervate formation may help to encapsulate invading virus particles, preventing their penetration across the mucus layer to the endothelial cell layer, and to facilitate the removal of the virus by the cilia-driven particle transport.

## 4. Materials and Methods

### 4.1. Materials

Na-polyphosphate (Na-polyP) with an average chain length of 40 P_i_ units (polyP_40_) came from the Chemische Fabrik Budenheim (Budenheim, Germany). Quercetin was purchased (#Q4951, Sigma-Aldrich, Taufkirchen, Germany), similar to dexamethasone 21-phosphate disodium salt (DEX-P; #D1159, Sigma-Aldrich, Taufkirchen, Germany), mucin from bovine submaxillary glands (type I-S) (#M3895, Sigma-Aldrich, Taufkirchen, Germany), thiazolyl blue tetrazolium bromide (MTT; #M2128, Sigma-Aldrich, Taufkirchen, Germany) and Ham’s F-12K (Kaighn’s) medium (#21127022, Gibco/Thermo Fisher Scientific, Dreieich, Germany).

### 4.2. Preparation of Mg-polyP Nanoparticles and Dexamethasone-Loaded Particles

Amorphous Mg-polyP nanoparticles (Mg-polyP-NP) were prepared as described [41,76], with modifications. Basically, the introduced procedure for the fabrication of Ca-polyP nanoparticles was applied [40]. A 2:1 molar ratio between MgCl_2_ and Na-polyP (based on phosphate) was used and the pH was adjusted to 10. For the reaction, 1 g of Na-polyP was dissolved in 100 mL of distilled water and 3.86 g of MgCl_2_∙6H_2_O (#A537.1, Roth, Karlsruhe, Germany) were dissolved in 100 mL. The MgCl_2_ solution was slowly and dropwise added to the polyP solution during a 60 min period of time. The pH was adjusted close to 10 (with NaOH). After stirring for 12 h, the nanoparticles (NP) were collected by filtration, washed twice with ethanol and then three-times with water and dried at 50 °C. The particles were collected; “Mg-polyP-NP”.

For loading of the particles with dexamethasone the corticosteroid was added to polyP at a mass concentrations of 5 wt% with respect to Na-polyP [77]. Dexamethasone 21-phosphate disodium salt (DEX-P) was used. Then, the polyP solution was supplemented with MgCl_2_ and processed as described. Finally the particles were collected, washed three times with water, and freeze-dried; “Mg-polyP/D-NP”.

### 4.3. Encapsulation Efficiency of Dexamethasone

The encapsulation efficiency of dexamethasone in the “Mg-polyP-NP” was determined spectrophotometrically by ultraviolet–visible spectroscopy (UV–Vis spectrometer; NanoDrop 2000c; Thermo Fisher Scientific, Dreieich, Germany). A calibration curve was established using DEX-P, as described [78], and the absorbance values at 242 nm within the concentration range (0–20 µg mL^−1^). The release kinetics from the NP was measured using 20 mg of “Mg-polyP/D-NP”, as described [77]. The particles were dissolved in 1 mL of HCl (0.5 M) and the solution was diluted with water while being vigorously vortexed for 10 min. After centrifugation (3000 rpm for 10 min) the concentration of dexamethasone was determined in the supernatant using the calibration curve. Following this procedure the percentage proportion of dexamethasone in the particles was 2.63 ± 0.5 µg per 100 µg of NP in the “Mg-polyP/D-NP” particles (n = 5 parallel experiments).

### 4.4. Preparation of the Quercetin-PolyP Nanoparticles

For the preparation of quercetin-polyP nanoparticles (Mg-polyP/QCT-NP) the “Mg-polyP-NP” were, after preparation, washed in an ethanolic QCT solution of 0.3 mg mL^−1^. In this step, 10 mL of the QCT solution was supplemented with 100 mg of “Mg-polyP-NP” and stirred for 60 min. Then, the particles were collected, washed with water and used for the experiments; “Mg-polyP/QCT-NP”.

### 4.5. Encapsulation Efficiency of QCT

In order to determine the QCT content in “Mg-polyP/QCT-NP”, the particles were suspended in citric acid (at pH 3.0) and incubated for 12 h at 37 °C. Then, the supernatant was collected, and the absorbance was measured at 366 nm [46,47,79,80]. A calibration curve was established and used for the assessment of the concentration of QCT in the particles. After this procedure the percentage proportion of QCT in the NP was determined to be 0.8 ± 0.04 µg per 100 µg of NP; in turn the yield of the compound during the reaction was 26%.

### 4.6. Interaction of “Mg-polyP-NP” with Mucin

Commercial mucin (from bovine submaxillary glands) was purified as described [81]. The fraction after differential centrifugation at a density between 1.45 g mL^−1^ and 1.49 g mL^−1^ was collected. Where indicated, an aliquot of 2 mg was subjected to 1 mL of 0.25 M NaCl and supplemented with 10 mg of “Mg-polyP-NP”.

### 4.7. QCT Release

The release kinetics of QCT from “Mg-polyP/QCT-NP” was determined in medium/serum. An aliquot of 10 mg of the particles was suspended in 5 mL of medium/serum; then, after the indicated time, the level of QCT was determined in the supernatant spectrophotometrically.

### 4.8. Microscopic Analyses

The scanning electron microscopic (SEM) images were performed with a HITACHI SU8000 microscope (Hitachi, Krefeld, Germany).

### 4.9. Particle Size Determination

The Zetasizer Nano ZS90 (Malvern Instruments, Malvern, UK) was used to determine the size of the particles by light scattering [82,83]. The particles were dispersed in ultrapure water.

### 4.10. Fourier Transformed Infrared Spectroscopy

Fourier transformed infrared spectroscopy (FTIR) was carried out with an ATR (attenuated total reflectance)-FTIR spectroscope/Varian 660-IR spectrometer (Agilent, Santa Clara, CA, USA), fitted with a Golden Gate ATR unit (Specac, Orpington, UK). The samples were pressed to small discs and subjected to the determination.

### 4.11. A549 Cells

A549 cells (#86012804, Sigma-Aldrich, Taufkirchen, Germany), a human lung (carcinoma) line, were cultivated in Ham’s F-12K (Kaighn’s) medium, supplemented with 10% fetal bovine serum, 1% penicillin-streptomycin, and 4 mM glutamine. The cells were incubated in 96-well plates or 24-well plates in a humidified atmosphere of 5% CO_2_ in air (37 °C) as described [84]. Every 3 to 4 d the medium/serum was replaced. If the cells were incubated with additional compounds for a longer period of time, half of the medium/serum was replaced after 3 d with new medium/serum containing the original components with the concentration used for the inoculum.

The incubation with polyP, both Na-polyP or the indicated Mg-polyP formulation, either the one-component “Mg-polyP-NP”, or this preparation together with dexamethasone “Mg-polyP/D-NP” or QCT “Mg-polyP/QCT-NP” at the indicated concentration was added. In the series with Na-polyP additional Mg^2+^ (molar ratio of 2 Mg^2+^ with respect to phosphate monomer, in the polyP) was added to the assays in order to compensate for the complexation potential of the polyanionic polymer [85].

### 4.12. MTT Metabolic Activity Assay

The viability kinetics of the cells was assessed by the determination of the metabolic activity, using the MTT assay [86]. The incubation period was 0 or 3 days. After termination, the cells were incubated first with MTT (1 μg mL^−1^; 2 h) and subsequently with 20% SDS in 50% dimethyl-formamide for 24 h [87]. The formazan grains were dissolved followed by the determination of the optical density 595 nm. Ten parallel experiments were performed.

### 4.13. Quantitative Real-Time Polymerase Chain Reaction

A detailed description of the qRT-PCR procedure has been given before [41]. The A549 cells were exposed to either “Mg-polyP-NP” or dexamethasone or quercetin alone or to the caged fraction “Mg-polyP/D-NP” or “Mg-polyP/QCT-NP” at concentrations given in the results. The cells were seeded at a density of 7 × 10^3^ cells mL^−1^ medium/serum and incubated for 3 and 6 d, respectively. Then, the cells were harvested, and RNA was extracted and used for the qRT-PCR reaction. The following primer pairs were used: for human *MUC5AC* [35] (U06711) Fwd: 5′-TCCGGCCTCATCTTCTCC-3′ and Rev: 5′-ACTTGGGCACTGGTGCTG-3′. After termination of the reaction, the expression levels were correlated with the one of the reference housekeeping gene *GAPDH* (glyceraldehyde 3-phosphate dehydrogenase; NM_002046.3) with the primer pair Fwd: 5′-ACTTTGTGAAGCTCATTTCC-3′ and Rev: 5′-TTGCTGGGGCTGGTGGTCCA-3′. The amplifications were performed in triplicate in an iCycler (Bio-Rad, Hercules, CA, USA), and the mean C_t_ values and the efficiencies were calculated applying the iCycler software (Bio-Rad) [88]; the estimated PCR efficiencies were 95–103%.

### 4.14. Immunofluorescence Analysis

After termination of the experiments after a 3 d incubation period, the A549 cells were washed in PBS, fixed and permeabilized with methanol/EGTA for 10 min [89]. Then, the cells were incubated with anti-*MUC5AC* antibodies (#HPA040615; Sigma-Aldrich, Taufkirchen, Germany, produced in rabbit); 1:1000 dilution in blocking reagent. Subsequently the samples were reacted with goat anti-rabbit IgG secondary antibodies (Alexa Fluor Plus 647; Thermo Fisher Scientific, Dreieich, Germany). The specimens were inspected with an Olympus AHBT3 microscope.

### 4.15. Statistical Analysis

The skewness of the non-Gaussian distribution curves of the NP was evaluated as published [82,83,90]. The values represent the respective average diameter ± standard deviations (σ). Student-*t* test was applied to perform comparisons between two groups. Usually, the average values and σ originated from at least three to six independent experiments. Values of *p* < 0.05 were considered statistically significant (*). The calculations were performed with the GraphPad Prism 7.0 software (GraphPad Software, La Jolla, CA, USA).

## 5. Conclusions

**Figure 10:**
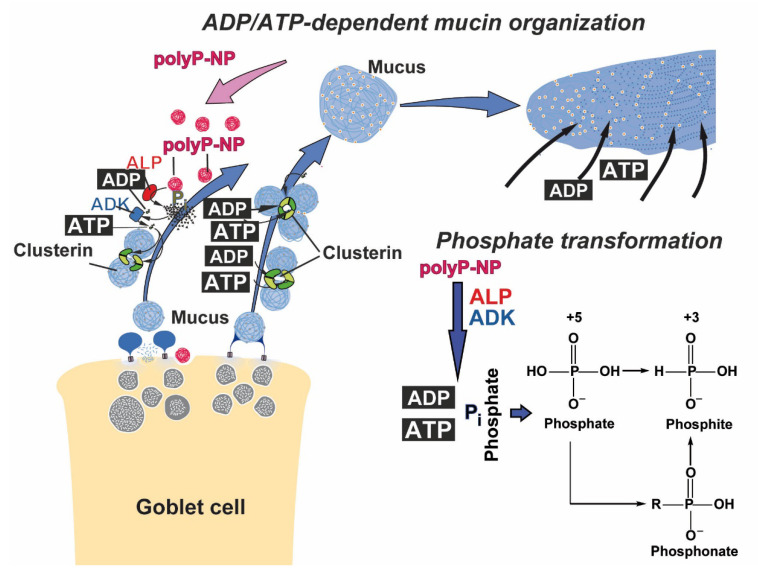
A proposed scheme summarizing the concept that ATP, or its lower-energy precursor ADP, feeds heat shock protein(s), or the chaperone-related molecule clusterin, in the mucin layers, driving the association of the mucins to an organized composition. Mucus is released from the goblet cells and—after the explosive exocytic discharge and the extensive absorption of water, is organized to a homogeneous net-like web. In this scheme, it is postulated that it is clusterin that organizes the individual mucin molecules. The embedded scheme shows the transition of phosphate to the reduced phosphite in a reversible redox reaction.

The present study shows that caged NP formed of polyP with dexamethasone and QCT display morphogenetic activity and cause an increased expression of mucin, as demonstrated here with *MUC5AC*. One common mode of action might be the fact that all three components are able to scavenge free oxygen species. This property has also been proposed for phosphate that is released during the ALP-mediated enzymatic cleavage of polyP; at least some metal salts (Mn^2+^) of phosphate may inactivate oxygen radicals [72]. It might also be possible that these phosphate molecules elicit antioxidant activity because of the existing redox cycle between phosphate and phosphite with the intermediate phosphonate (Figure 10). However, a metabolic formation of phosphite from phosphonates has not yet been demonstrated [91]. Phosphonate C-P bonds could be detected in sponges [58] and even humans [92], but it seems more likely that they originate from associated bacteria or food. On the other hand, enzymatic oxidation of phosphite to phosphate proceeds in some bacteria. Interestingly, the phosphoryl transfer reaction catalyzed by the bacterial phosphite-NAD^+^ oxidoreductase seems to involve the formation of a metaphosphate intermediate [93], as proposed for the formation of ADP from polyP by the ALP [17]. Besides the morphogenetic activities of the three components, the release of ATP during ALP degradation of the caged NP in the concert with the mucin expression [94] also has a potential organizing effect on the mucin layer on the surface of the endothelial cells of the airways system; Figure 10. While ATP certainly has a determining effect on goblet cells that produce the mucins, it might also have a structure-giving and form-formatting function during the association processes of the mucin molecules after the explosive exocytic release of the mucin granules [95], as shown in Figure 10. Future studies must prove the view (see “Introduction”) that ATP or its lower-energy precursor ADP control this association and organization of the mucus layer via heat shock proteins or the chaperone-related molecule clusterin.

## Data Availability

The data presented in this study are available on request from the corresponding authors.
